# Prognostic value of NT-proBNP levels in the acute phase of sepsis on lower long-term physical function and muscle strength in sepsis survivors

**DOI:** 10.1186/s13054-019-2505-7

**Published:** 2019-06-24

**Authors:** Carlo Custodero, Quran Wu, Gabriela L. Ghita, Stephen D. Anton, Scott C. Brakenridge, Babette A. Brumback, Philip A. Efron, Anna K. Gardner, Christiaan Leeuwenburgh, Lyle L. Moldawer, John W. Petersen, Frederick A. Moore, Robert T. Mankowski

**Affiliations:** 1 Department of Aging and Geriatric Research, University of Florida, 2004 Mowry Road, Gainesville, FL 32611 USA; 2 Dipartimento Interdisciplinare di Medicina, Clinica Medica Cesare Frugoni, University of Bari Aldo Moro, Bari, Italy; 3 Department of Surgery, University of Florida, Gainesville, FL USA; 4 Department of Biostatistics, University of Florida, Gainesville, FL USA; 5 Department of Cardiology, University of Florida, Gainesville, FL USA

**Keywords:** Sepsis, Prognostic value, N-terminal pro-brain natriuretic peptide, Short physical performance battery, Hand grip strength

## Abstract

**Background:**

Sepsis survivors often develop chronic critical illness (CCI) and demonstrate the persistent inflammation, immunosuppression, and catabolism syndrome predisposing them to long-term functional limitations and higher mortality. There is a need to identify biomarkers that can predict long-term worsening of physical function to be able to act early and prevent mobility loss. N-terminal pro-brain natriuretic peptide (NT-proBNP) is a well-accepted biomarker of cardiac overload, but it has also been shown to be associated with long-term physical function decline. We explored whether NT-proBNP blood levels in the acute phase of sepsis are associated with physical function and muscle strength impairment at 6 and 12 months after sepsis onset.

**Methods:**

This is a retrospective analysis conducted in 196 sepsis patients (aged 18–86 years old) as part of the University of Florida (UF) Sepsis and Critical Illness Research Center (SCIRC) who consented to participate in the 12-month follow-up study. NT-proBNP was measured at 24 h after sepsis onset. Patients were followed to determine physical function by short physical performance battery (SPPB) test score (scale 0 to12—higher score corresponds with better physical function) and upper limb muscle strength by hand grip strength test (kilograms) at 6 and 12 months. We used a multivariate linear regression model to test an association between NT-proBNP levels, SPPB, and hand grip strength scores. Missing follow-up data or absence due to death was accounted for by using inverse probability weighting based on concurrent health performance status scores. Statistical significance was set at *p ≤* 0.05.

**Results:**

After adjusting for covariates (age, gender, race, Charlson comorbidity index, APACHE II score, and presence of CCI condition), higher levels of NT-proBNP at 24 h after sepsis onset were associated with lower SPPB scores at 12 months (*p* < 0.05) and lower hand grip strength at 6-month (*p* < 0.001) and 12-month follow-up (*p* < 0.05).

**Conclusions:**

NT-proBNP levels during the acute phase of sepsis may be a useful indicator of higher risk of long-term impairments in physical function and muscle strength in sepsis survivors.

## Background

Sepsis is a major public health problem, worldwide and according to the Center of Disease Control (CDC); around 1.7 million adults in the USA become septic each year [1]. Sepsis is diagnosed in 6% of hospitalized patients and costs approximately $20 billion annually [1]. Although there have been advances in early diagnosis and treatment with a significant reduction of in-hospital mortality, many survivors develop chronic critical illness (CCI) and the persistent inflammation, immunosuppression, and catabolism syndrome predisposing them to long-term organ dysfunction, chronic inflammation, functional limitations, and higher mortality [2, 3]. Studies have shown that progressive decline in physical function after critical illness, such as sepsis, leads to disability, dependence on caregivers, and higher long-term mortality [4–6]. Therefore, identification of acute-phase biomarkers associated with poor long-term physical function in sepsis survivors could serve as an indicator for early implementation of strategies to prevent functional decline and disability.

We know that sepsis insult affects many organs and contributes to the long-term exacerbation of cardiovascular dysfunction [7–9] and skeletal muscle wasting [10]. Physiologically, impaired cardiovascular function resulting in decreased peripheral blood perfusion, lower oxygenation, and nutrient delivery may contribute to muscle wasting and physical dysfunction, long-term. Therefore, cardiovascular dysfunction in the acute phase of sepsis may be indicative of long-term physical dysfunction.

N-terminal pro-brain natriuretic peptide (NT-proBNP) is a cardiac-overload biomarker associated with cardiovascular disease severity [11] and sepsis severity [12] but also with long-term physical function decline after cardiothoracic surgery [13]. NT-proBNP is an inactive fragment of the neurohormone brain natriuretic peptide (BNP). BNP and NT-proBNP are released into the bloodstream by cardiomyocytes in response to stress and pressure posed on the atria and ventricles. Usually, patients suffering from sepsis show signs of cardiac insufficiency [14]. Indeed, the destructive systemic inflammatory response and intensive resuscitation occurring during sepsis result in widespread organ overload and damage including myocardial injury [15]. Additionally, experimental studies have demonstrated that endotoxins and cytokines directly increase gene expression of BNP, explaining the higher levels of this biomarker during sepsis [16]. Growing evidence shows that NT-proBNP may represent a useful prognostic biomarker in septic patients [17–20].

Noteworthy, elevated NT-proBNP has been associated with an almost sevenfold higher risk of mortality in sepsis [20] and is a predictor of survival when compared to traditional biomarkers of sepsis including C-reactive protein and procalcitonin [21–23]. On the other hand, septic cardiac dysfunction in the acute phase has been suggested to be a protective mechanism of cardiomyocytes against the cytokine surge and suggested to be fully reversible after 7–10 days [24]; however, it is unclear whether this temporary cardiovascular dysfunction during sepsis may result in long-term consequences on physical function and muscle strength impairment.

A possible relationship between acute NT-proBNP levels, objective long-term physical function, and muscle strength measures has not been studied in a sepsis population. Therefore, the aim of the present study was to test whether higher 24-h NT-proBNP levels are associated with worse physical function and hand grip strength at 6 and 12 months after sepsis.

## Methods

### Study design and population

This is a retrospective analysis of the longitudinal cohort study carried out from January 2015 to April 2018 by the Sepsis and Critical Illness Research Center (SCIRC) at the University of Florida (UF) (Gainesville, FL, USA). The purpose of the over parent study was to look at the long-term consequences of sepsis in humans and to better understand the biological mechanisms of the septic insult animals models. The study included 224 adult patients (age ≥ 18 years) from trauma and surgical intensive care units (ICUs) who developed sepsis meeting Sepsis-2 consensus definitions [25]. Subjects were excluded for one of the following criteria: age < 18 years, severe traumatic brain injury (i.e., computed tomography evidence of neurological injury and Glasgow Coma Scale score < 8), permanent sensory and/or motor deficit from spinal cord injury, sepsis with an uncontrollable source (e.g., unresectable bowel ischemia), New York Heart Association class IV heart failure, Child-Pugh class B or C liver disease, known HIV infection with CD4 count < 200 cells/mm, organ transplant recipient on an immunosuppressant agent, chemotherapy or radiotherapy within 30 days prior to onset of sepsis, reduced lifespan (< 3 months) for pre-existing comorbidities, active end-of-life decision (do not resuscitate/do not intubate), pregnancy, incarceration, or institutionalization. We analyzed a subsample of 196 consecutive sepsis patients who consented to participate in the 12-month follow-up study and who had 24-h NT-proBNP levels. The study has been registered at ClinicalTrials.gov (NCT02276417).

### Ethics

This study was approved by the University of Florida’s Institutional Review Board. Patient (if able) or legally authorized representative provided written informed consent within 96 h after the patient qualified for the inclusion in the study. If consent was not obtained within 96 h, all bio-specimens and patient data were destroyed. If consent was initially obtained from the legally authorized representative and the patient regained decision-making capacity, the patient could withdraw from the study.

### Assessment procedures

All patients were initially screened every 4 h using the Modified Early Warning Signs - Sepsis Recognition Score (MEWS-SRS). The overall MEWS-SRS score is the sum of worsening individual scores (0 to 3) for temperature, heart rate, respiratory rate, systolic blood pressure, change in mental status (MS), and most recent white cell count [26]. Patients with MEWS-SRS > 6 (or 5 when MS cannot be assessed) underwent a secondary screen by a physician or advanced care practice provider who specifically evaluates for the presence of sepsis, severe sepsis, or septic shock according to consensus definitions [25, 27–29]. Subsequently, the patient records were adjudicated by the clinical faculty members of the SCIRC at weekly SCIRC sepsis adjudication meetings to ensure the appropriate diagnosis of sepsis and its severity. Patients who were eligible for the study were entered into a standardized evidenced-based protocol designed to meet the Surviving Sepsis Campaign. Details documenting high protocol compliance have been described elsewhere [30]. Baseline demographic data was collected including age, gender, race, body mass index (BMI), Charlson comorbidity index (calculating burden of chronic illnesses) [31], Acute Physiology and Chronic Health Evaluation (APACHE) II score at 24 h (which measures disease severity) [32], ICU and hospital length of stay (LOS), and discharge disposition. Patients were categorized into three groups: (a) early deaths (< 14 days), (b) RAP defined as staying in the ICU for < 14 days, and (c) CCI defined as ICU stay for ≥ 14 days with evidence of ongoing organ dysfunction as assessed by a Sequential Organ Failure Assessment (SOFA) score on day 14 of at least two in any organ system or at least one for cardiovascular system [3].

### Sample collection and testing

Blood samples were collected from septic patients at 24 h after sepsis onset. Blood samples were immediately put on ice and processed within 30 min. Thereafter, obtained serum samples were kept frozen at − 80 °C. NT-proBNP levels were determined using the quantitative electrochemiluminescent immunoassay (ECLIA) method on an Elecsys 2010 (Roche Diagnostics) analyzer. The measuring range of NT-proBNP was 5–35,000 ng/L [33]. According to the 2012 European Society of Cardiology guidelines for heart failure, age-independent cutoff levels for NT-proBNP of ≤ 300 ng/L exclude acute heart failure [34].

### Outcomes

Physical function and muscle strength were evaluated at 6 and 12 months after sepsis onset in CCI and RAP survivors by a trained research coordinator at the UF Institute on Aging. The testing was performed at the patient’s home, if the patients were unwilling or unable to come to the clinic for follow-up visits. Physical function was assessed objectively by short physical performance battery (SPPB). SPPB consists of three items: 4-m walk, repeated chair stands, and three increasingly difficult standing balance tests. Each task received a score from 0 (worst performance) to 4 (best performance). The total score ranging from 0 to 12 was computed by summing the three component scores [35].

Muscle strength was assessed by hand grip strength test. It was measured in kilograms using a hand-held dynamometer (Jamar Hydraulic Hand Dynamometer, Model No. BK-7498; Fred Sammons, Burr Ridge, IL). The dynamometer was individually adjusted for hand size, and two trials were performed for each hand. The subjects were asked to squeeze the dynamometer as hard as possible twice [36].

Alternatively, for patients missing follow-up visits, information regarding performance status was collected using Zubrod score estimates based on qualitative notes taken during monthly phone calls. The Zubrod scale is a 5-point scale which measures the performance status ranging from 0 to 5, with 0 denoting perfect health, 1 for symptomatic subjects but able to carry out normal daily activities, 2 for symptomatic subjects who were in bed ≤ 50% of daytime, 3 for symptomatic subjects who were in bed for more than half of daytime, 4 for completely bedridden subjects, and 5 denoting death [6].

### Statistical analysis

Data was presented as either frequency and percentage for categorical variables or mean and standard deviation (SD), median, and interquartile range (IQR) for continuous variables. Fisher’s exact test was used for comparison of categorical variables, while *t* test and Kruskal-Wallis test were used for comparison of mean and median of continuous variables, respectively. Multivariate linear regression was constructed to determine independent associations between 24-h NT-proBNP levels, SPPB score, and hand grip strength at 6 months and 12 months. Variables entered into the model were defined a priori. NT-proBNP was categorized by tertiles (lowest, 29–711 ng/L, considered as the reference group; middle, 712–2378 ng/L; highest, 2379–70,000 ng/L). Other variables included in the model were age, Charlson comorbidity index and APACHE II score assessed as continuous variables, gender, race (white Caucasian vs not white Caucasian), and presence of CCI condition assessed as dichotomized variables. Inverse probability weighting based on concurrent Zubrod scores was used to account for missing follow-up data, as well as absence due to death. All significance tests were performed as two-sided, with *p* ≤ 0.05 considered as statistically significant. All analyses were conducted under SAS 9.4 TS1M1 (SAS Institute Incorporated, Cary, NC, USA).

## Results

### Study patients

**Fig. 1:**
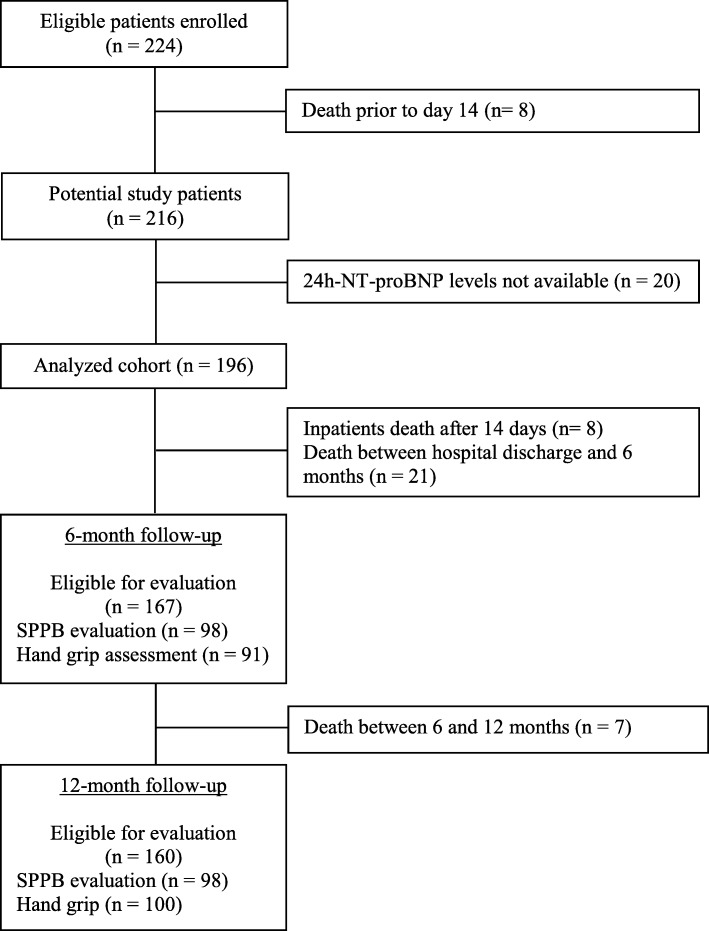
Consolidated Standards of Reporting Trials diagram and retention rates of a 12-month follow-up

Figure 1 shows the subjects eligible for evaluation at each step of the study process. Over 39 months ending April 2018, 224 patients were enrolled in the SCIRC sepsis database. There were 8 (3.6%) early deaths, leaving 216 potential study patients. A subsample of 196 consecutive subjects (91%) had 24-h NT-proBNP levels and was included in this focused study. Of these 196 patients, 122 (62%) were classified as RAP and 74 (38%) were classified as CCI. Of those classified as RAP, 3 (2.5%) died within 6 months, while another 3 (2.5%) died within 12 months, thus leaving 119 and 116 RAP patients potentially eligible for functional testing at 6 and 12 months, respectively. Of those classified as CCI, 26 (35%) died within 6 months, while 30 (41%) died within 12 months, leaving 48 and 44 subjects potentially eligible for functional testing at 6 and 12 months, respectively. At 6-month follow-up, 27 CCI and 71 RAP patients had SPPB assessed, while 22 CCI and 69 RAP patients provided hand grip strength measurements. At 12-month follow-up, 22 CCI and 76 RAP patients had SPPB score assessed, while 23 CCI and 77 RAP patients provided hand grip strength measurements.

### Baseline characteristics

One hundred ninety-six patients who had 24-h NT-proBNP levels were included in this analysis. Baseline characteristics of the study population are displayed in Table 1. The mean age of patients upon admission to the ICU was 59.1 ± 15.1. Overall, patients were more likely male (53.6%), white Caucasian (90.8%), and with a median BMI of 29 kg/m^2^ (IQR 24.8–35.6). The median ICU and hospital LOS overall were 8 (IQR 3.5–19) and 18 days (IQR 10–31), respectively. The mean Charlson comorbidity index at admission was 3.4 ± 2.8. On average, APACHE II score was 18 ± 8.1. Sixty patients (31%) were diagnosed with sepsis, 89 (45%) with severe sepsis, and 47 (24%) with septic shock. Among these patients, 74 (38%) developed a CCI condition with a median ICU length of stay (LOS) of 21 days (IQR 16–37) and a median hospital LOS of 31 days (IQR 22–47). Not surprisingly, CCI patients compared to the remaining 122 (62%) RAP patients had a greater Charlson comorbidity index, APACHE II scores, and percentage septic shock (36.5% vs 16.4%). In the whole sample, 44.4% of subjects were discharged to dispositions associated with poor outcomes (e.g., long-term acute care facility, skilled nursing facility, another hospital, or died), but among patients with CCI, this kind of discharge occurred more frequently compared to those with RAP (82.4% vs 21.3%, *p* < 0.0001). Median NT-proBNP level at 24 h after sepsis diagnosis was 1209 ng/L and was much higher in the CCI cohort (2219 ng/L vs RAP = 1084 ng/L, *p =* 0.0042).

**Table 1:** Baseline characteristics of the study population

	Overall (*n* = 196)	CCI (*n* = 74)	RAP (*n* = 122)
Age, mean (SD)	59.1 (15.1)	61.5 (14.4)	57.7 (15.5)
Male, *n* (%)	105 (53.6)	46 (62.2)	59 (48.4)
Race, *n* (%)			
White Caucasian	178 (90.8)	68 (91.9)	110 (90.2)
African American	15 (7.7)	5 (6.8)	10 (8.2)
Asian	1 (0.5)	0 (0)	1 (0.8)
Others	1 (0.5)	1 (1.4)	0 (0)
Unknown	1 (0.5)	0 (0)	1 (0.8)
BMI, median (IQR)	29 (24.8, 35.6)	29 (24.4, 36)	29.1 (24.9, 34.9)
Sepsis status, *n* (%)***			
Sepsis	60 (30.6)	11 (14.9)	49 (40.2)
Severe sepsis	89 (45.4)	36 (48.6)	53 (43.4)
Septic shock	47 (24)	27 (36.5)	20 (16.4)
Charlson comorbidity index, mean (SD)**	3.4 (2.8)	4 (2.8)	3 (2.8)
APACHE II score (24 h), mean (SD)***	18 (8.1)	21.4 (7.9)	15.9 (7.4)
CHF, *n* (%)*	22 (11.2)	13 (17.6)	9 (7.4)
Admitted for trauma, *n* (%)	18 (9.2)	10 (13.5)	8 (6.6)
NT-proBNP, ng/L, median (IQR)**	1209 (495, 4326)	2219 (707, 7585)	1084 (439, 2360)
Inter-facility hospital transfer, *n* (%)**	86 (43.9)	43 (58.1)	43 (35.2)
Hospital-acquired sepsis^a^, *n* (%)**	76 (38.8)	38 (51.4)	38 (31.1)
ICU LOS, median (IQR)***	8 (3.5, 19)	21 (16, 37)	5 (3, 8)
Hospital LOS, median (IQR)***	18 (10, 31)	31 (22, 47)	11.5 (7, 20)
Discharge disposition, *n* (%)			
“Good” disposition***	109 (55.6)	13 (17.6)	96 (78.7)
Home	34 (17.3)	1 (1.4)	33 (27)
Home healthcare services	66 (33.7)	9 (12.2)	57 (46.7)
Rehab	9 (4.6)	3 (4.1)	6 (4.9)
“Poor” disposition***	87 (44.4)	61 (82.4)	26 (21.3)
Long-term acute care facility	32 (16.3)	31 (41.9)	1 (0.8)
Skilled nursing facility	33 (16.8)	8 (10.8)	25 (20.5)
Another Hospital	9 (4.6)	9 (12.2)	0 (0)
Hospice	5 (2.6)	5 (6.8)	0 (0)
Death	8 (4.1)	8 (10.8)	0 (0)

*CCI* chronic critical illness, *RAP* rapid recovery, *SD* standard deviation, *BMI* body mass index, *IQR* interquartile range, *APACHE* Acute Physiology and Chronic Health Evaluation, *CHF* congestive heart failure, *NT-proBNP* N-terminal pro-brain natriuretic peptide, *ICU* intensive care unit, *LOS* length of stay

*p < 0.05 between the two groups, **p < 0.01 between the two groups, ***p < 0.001 between the two groups

^a^Sepsis onset ≥ 48 h after hospital admission

### Outcomes and CCI

**Fig. 2:**
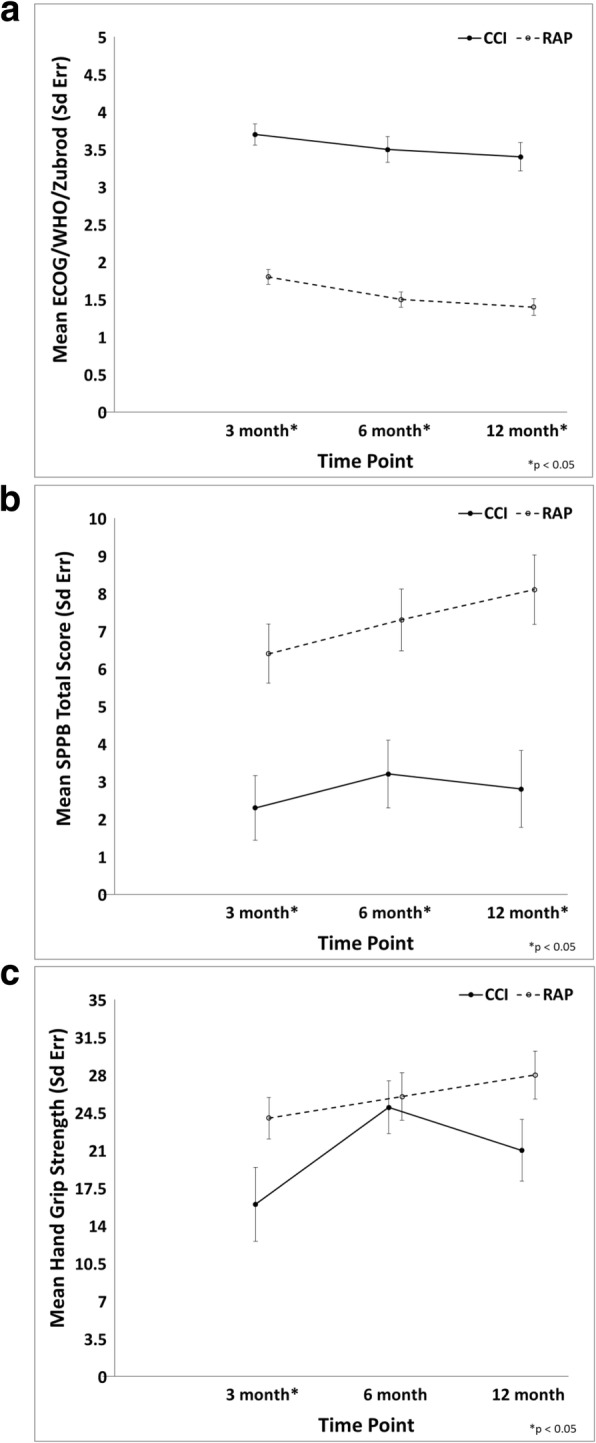
Line graphs of Zubrod score (**a**), SPPB (**b**), and hand grip strength (**c**) over time between CCI and RAP patients. CCI, chronic critical illness; RAP, rapid recovery; Sd Err, standard error. **a** ECOG/WHO, Eastern Cooperative Oncology Group/World Health Organization. **b** SPPB, short physical performance battery. The data were expressed as mean and standard error with statistical significance set at *p* < 0.05. Asterisk indicates a statistically significant difference

Functional testing at 6 and 12 months are depicted in Table 2. Weighted mean SPPB score and hand grip strength at 6 months were respectively 5.8 ± 0.69 and 26.1 ± 1.78 kg. The same tests were performed after 12 months from sepsis onset (weighted mean SPPB 6.2 ± 0.87, weighted mean hand grip strength 25.8 ± 1.89 kg). Septic patients who developed the CCI condition showed also the worsening of the Zubrod score from baseline and higher scores at 6 and 12 months compared to RAP patients (Table 2, Fig. 2a). Compared to patients who rapidly recovered, those who suffered from the CCI condition had a significantly lower SPPB score at 6 months (3.2 ± 0.9 vs 7.3 ± 0.82, *p* = 0.0011) and 12 months (2.8 ± 1.02 vs 8.1 ± 0.92, *p* = 0.0002) (Table 2, Fig. 2b). Although, no significant differences were found in the hand grip strength, at both visits, between RAP and CCI patients, a non-significant trend was observed for lower hand grip strength at 12 months in the CCI group compared to RAP (21.6 ± 2.88 vs 28 ± 2.22, *p* = 0.08) (Table 2, Fig. 2c).

**Table 2:** Physical performance and strength at 6 and 12 months in sepsis survivors

	Overall	CCI	RAP	*p* value
6-month visit				
SPPB score*, mean (SE)	5.8 (0.69)	3.2 (0.9)	7.3 (0.82)	0.0011
	(*n* = 98)	(*n* = 27)	(*n* = 71)	
Hand grip strength (kg)*, mean (SE)	26.1 (1.78)	25.4 (2.44)	26.3 (2.2)	0.77
	(*n* = 91)	(*n* = 22)	(*n* = 69)	
Zubrod, mean (SE)	2.2 (0.13)	3.4 (0.19)	1.5 (0.12)	< 0.0001
	(*n* = 174)	(*n* = 66)	(*n* = 108)	
12-month visit				
SPPB score*, mean (SE)	6.2 (0.87)	2.8 (1.02)	8.1 (0.92)	0.0002
	(*n* = 100)	(*n* = 24)	(*n* = 76)	
Hand grip strength (kg)*, mean (SE)	25.8 (1.89)	21.6 (2.88)	28 (2.22)	0.08
	(*n* = 100)	(*n* = 23)	(*n* = 77)	
Zubrod, mean (SE)	2.1 (0.14)	3.3 (0.22)	1.3 (0.14)	< 0.0001
	(*n* = 169)	(*n* = 64)	(*n* = 105)	

*SPPB* short physical performance battery, *SE* standard error, *CCI* chronic critical illness, *RAP* rapid recovery

*Inverse probability weighting based on concurrent Zubrod scores was used to account for missing follow-up data, as well as absence due to death

Tables 3 and 4 show the results from a multivariate generalized linear model including NT-proBNP, age, gender, race, Charlson comorbidity index, APACHE II score, and presence of CCI for the association with hand grip strength and SBBP at 6 and 12 months. CCI was independently associated with worse SPPB score both at 6-month (coefficient − 3.32, *p* = 0.0023) and 12-month (coefficient − 4.33, *p* < 0.0001) follow-up (Table 4). Presence of the CCI condition showed a negative but a non-significant association with hand grip strength at 6 (coefficient − 3.81, *p* = 0.099) and 12 (coefficient − 4.00, *p* = 0.0617) months, suggesting that patients with the CCI condition had a lower muscle strength in a long-term follow-up compared to RAP (Table 3).

**Table 3:** Multivariable linear regression model for an association between 24-h NT-proBNP levels and hand grip strength at 6 and 12 months after sepsis*

Effect	Estimate	SE	*p* value
6-month hand grip strength			
Intercept	57.18	3.86	< 0.0001
NT-proBNP (lowest tertile as reference)			0.0015^†^
Middle tertile	− 1.63	2.02	0.4222
Highest tertile	− 9.30	2.60	0.0006
Sex (female)	− 16.18	1.88	< 0.0001
Race (non-Caucasian)	2.80	3.41	0.4138
Charlson comorbidity index	− 0.09	0.52	0.8588
Age	− 0.32	0.09	0.0003
CCI^‡^	− 3.81	2.29	0.0990
APACHE	− 0.03	0.12	0.8372
12-month hand grip strength			
Intercept	56.87	5.21	< 0.0001
NT-proBNP (lowest tertile as reference)			0.0427^†^
Middle tertile	− 1.20	2.23	0.5916
Highest tertile	− 8.71	3.61	0.0177
Sex (female)	− 15.61	2.21	< 0.0001
Race (non-Caucasian)	3.65	2.83	0.2010
Charlson comorbidity index	− 0.05	0.62	0.9380
Age	− 0.31	0.12	0.0146
CCI^‡^	− 4.00	2.11	0.0617
APACHE	− 0.07	0.11	0.5386

Inverse probability weighting based on concurrent Zubrod scores was used to account for missing follow-up data, as well as absence due to death; NT-proBNP levels: lowest tertile (29–711 ng/L), middle tertile (712–2378 ng/L), and highest tertile (2379–70,000 ng/L)

*SE* standard error, *NT-proBNP* N-terminal pro-brain natriuretic peptide, *CCI* chronic critical illness, *APACHE* Acute Physiology and Chronic Health Evaluation

^†^Overall *p* value

^‡^Rapid recovery (RAP) patients as a reference group

**Table 4:** Multivariable linear regression model for an association between 24-h NT-proBNP levels and physical performance on the SPPB at 6 and 12 months after sepsis

Effect	Estimate	SE	*p*
6-month SPPB			
Intercept	11.28	2.10	< 0.0001
NT-proBNP (lowest tertile as reference)			0.0118^†^
Middle tertile	1.25	1.11	0.2596
Highest tertile	− 2.24	1.50	0.1378
Sex (female)	− 1.51	0.93	0.1069
Race (non-Caucasian)	− 0.62	1.38	0.6512
Charlson comorbidity index	− 0.61	0.19	0.0014
Age	− 0.03	0.04	0.4986
CCI^‡^	− 3.32	1.06	0.0023
APACHE	0.03	0.06	0.6476
12-month SPPB			
Intercept	12.89	1.66	< 0.0001
NT-proBNP (lowest tertile as reference)			0.0060^†^
Middle tertile	0.02	0.90	0.9825
Highest tertile	− 2.92	1.20	0.0165
Sex (female)	− 2.79	0.71	0.0002
Race (non-Caucasian)	− 1.01	1.07	0.3487
Charlson comorbidity index	− 0.75	0.12	< 0.0001
Age	− 0.009	0.04	0.8206
CCI^‡^	− 4.33	0.88	< 0.0001
APACHE	0.04	0.04	0.3579

Inverse probability weighting based on concurrent Zubrod scores was used to account for missing follow-up data, as well as absence due to death; NT-proBNP levels: lowest tertile (29–711 ng/L), middle tertile (712–2378 ng/L), and highest tertile (2379–70,000 ng/L)

*SPPB* short physical performance battery, *SE* standard error, *NT-proBNP* N-terminal pro-brain natriuretic peptide, *CCI* chronic critical illness, *APACHE* Acute Physiology and Chronic Health Evaluation

^†^Overall *p* value

^‡^Rapid recovery (RAP) patients as a reference group

### NT-proBNP and long-term physical function and strength

Multivariate analysis showed that NT-proBNP levels were significantly associated with grip strength at 6 months (*p* = 0.0015) and at 12 months (*p* = 0.0427), after adjusting for covariates, i.e., age, sex, race, Charlson comorbidity index, APACHE II score, and presence of CCI condition (Table 3). In particular, higher NT-proBNP levels (> 2378 ng/L) were related to a reduction of grip strength by 9.3 kg at 6 months (*p* = 0.0006) and by 8.71 kg at 12 months (*p* = 0.0177). After adjusting for the covariates, NT-proBNP levels were significantly associated with the SPPB score at 6 (overall *p* = 0.0118 across tertiles) and at 12 (overall *p* = 0.0060 across tertiles) months. However, while at 12-month follow-up, higher NT-proBNP levels were related with lower SPPB scores (coefficient − 2.92, *p* = 0.0165), no significant difference was found between subjects in the highest tertile compared to those who were in the lowest one at 6 months (Table 4).

## Discussion

The main finding of this analysis is that NT-proBNP levels measured at 24 h after sepsis onset were associated with worse physical function and upper limb muscle strength at 6 and 12 months after sepsis.

In agreement with previous work of our group, the presence of CCI condition may predispose to dismal outcomes in sepsis survivors [3, 6]. Accordingly, in this analysis, we found that CCI condition was indeed associated with poorer baseline characteristics, higher age, poorer discharge disposition, and higher levels of NT-proBNP compared to patients who rapidly recovered (RAP). At both 6- and 12-month follow-up time points, physical function but not muscle strength was significantly lower among CCI patients.

Potential mechanisms explaining the increased NT-proBNP levels during sepsis are still debated. The most accepted hypothesis is that cardiac insufficiency during sepsis is caused by the impact of the pro-inflammatory response and ventricular overload during aggressive fluid resuscitation therapy [15, 37]. Current literature argues that sepsis-induced cardiac dysfunction is a reversible condition driven by functional rather than structural changes, with a complete resolution within 7–10 days in survivors [24]. However, recently, Khoury et al. demonstrated that plasma BNP levels at ICU admission were predictive of 90-day and 60-month all-cause mortality in septic patients without heart failure [38]. Thus, despite their short half-life, BNP and NT-proBNP may have relevance in the long-term prognosis of sepsis survivors.

The novelty of our findings is that NT-proBNP levels were associated with objectively measured long-term physical function and muscle strength. In particular, we examined SPPB and upper limb muscle strength measured by hand grip strength test at 6 and 12 months after sepsis onset, which have been shown as highly relevant predictors of disability, institutionalization, and mortality [39–41].

Sporadic evidence about a relationship between natriuretic peptides, physical function, and muscle mass originated from studies on patients with coronary artery disease and healthy subjects. For example, Fox et al., in a cohort of patients undergoing coronary artery bypass graft (CABG) surgery, showed that peak plasma BNP levels, measured between 1 and 5 postoperative days, were significantly related to physical function decline at 6, 12, and 24 months after surgery [13]. Noteworthy, they demonstrated that physical function levels did not differ between 6 and 12 months after surgery, but the physical function levels declined significantly between 12 and 24 months [13]. In the study by Fox et al., CABG patients were more likely to have structural cardiac abnormalities and thus get some benefit on physical function within 12 months after surgery. Also, the authors measured physical performance using the Short Form-36 (SF-36) physical function domain, a questionnaire in which patients self-reported their levels of physical activity; thus, it may be a less reliable assessment of physical function levels. In our analysis, subjects with 24-h levels of NT-proBNP higher than 2378 ng/L showed poorer grip strength since 6-month follow-up, and significantly lower SPPB score at 12 months, but not at 6 months. We can only speculate that, since the SPPB score appears also strictly related to CCI condition, physical function decline could be secondary to the cardiovascular dysfunction that develops over time. However, we also cannot exclude a potential effect of exacerbation of comorbid conditions or readmissions. In another study, Yamashita et al. demonstrated that plasma BNP was significantly and negatively correlated with skeletal muscle mass in middle-aged and older adults, such that sarcopenic individuals had higher BNP levels [42].

It is well accepted that the ICU-acquired weakness (ICU-AW) is clinically characterized by bilateral and symmetrical limb weakness [43]. This may be a manifestation of a rising catabolic state in septic patients who fail to rapidly recover from an acute event. Findings from animal models show that sepsis triggers severe and sustained muscle fiber atrophy in the limb muscles, which is associated with enhanced proteasomal and autophagic proteolytic pathway activities, increased mitochondrial injury, and reduced electrical excitability in the skeletal muscle membrane [44–46]. Furthermore, the long-term effect of ICU-AW might also be driven by an impaired muscle regeneration due to satellite cell dysfunction [47]. In a shorter follow-up period compared to our study, Solverson et al. showed that after 3 months from hospital discharge, survivors of critical illness had reduced hand grip strength and 6-min walk distance in association with the presence of sepsis but not ICU LOS [48].

In line with these data, we found that higher NT-proBNP levels had negative prognostic value on long-term hand grip strength and SPPB scores independently of the presence of CCI condition, which implies a longer ICU LOS. Moreover, such a correlation was also independent of other potential confounders including age, gender, race, comorbidities, and illness severity. Therefore, the cardiac stress and overload occurring during sepsis might sustain the long-term limitation in physical functions, and maybe in the next future, NT-proBNP might represent a useful prognostic marker.

There were a number of strengths of the present study. First, data was obtained from a unique cohort of sepsis patients using a prospective, longitudinal design with in-person and phone follow-up. Additionally, this study included an intense assessment using multiple measures (both subjective and objective) to assess physical function. Finally, our high retention rates at follow-up visits have provided new information about trajectories following sepsis, especially in the extremely low-functioning CCI patient population.

### Limitations

There are also limitations to this study. First, our study was conducted in a retrospective fashion in a specific cohort of trauma and surgical patients in the ICU, including only those individuals who had 24-h NT-proBNP levels. Thus, the sample may not be a representative of the broader population of sepsis survivors, and this may limit our ability to make a generalization. Second, we did not have information about physical functions of patients before ICU admission, trauma to the upper and lower limbs at admission, and eventual readmission during follow-up which might influence the performances. However, only a small fraction of the cohort suffered trauma (9.2%). Third, there was a larger variation of NT-proBNP levels across the study sample. It is conceivable that both aggressive fluid management in cases of septic shock and use of medications with potential cardiotoxic effect may have led to NT-proBNP elevation.

### Future directions

A deeper understanding of the pathophysiological mechanisms of long-term cardiovascular consequences of sepsis and further investigation of the reliability of NT-proBNP as a sepsis biomarker could offer opportunities to improve therapeutic strategies and reduce risk of functional limitations among sepsis survivors. Future studies warrant investigating the associations between different ranges of elevated NT-proBNP levels, levels of physical function impairment and cardiovascular function, and longer follow-up. Since NT-proBNP is a simple and relatively cost-effective biomarker, future work should include assessment of NT-proBNP in sepsis survivors at higher risk of long-term poor functional outcomes.

## Conclusions

This study suggests that 24-h NT-proBNP levels were associated with worse long-term muscle strength and physical function among sepsis survivors. Future studies are warranted to further test the value of acute NT-proBNP levels as an indicator of long-term physical disability.

## Data Availability

The dataset used during the current study is available from the corresponding author on reasonable request.

## Abbreviations

APACHE: Acute Physiology and Chronic Health Evaluation
BMI: Body mass index
BNP: Brain natriuretic peptide
CABG: Coronary artery bypass graft
CCI: Chronic critical illness
CDC: Center of Disease Control
CHF: Congestive heart failure
ICU: Intensive care unit
ICU-AW: Intensive care unit-acquired weakness
IQR: Interquartile range
LOS: Length of stay
MEWS-SRS: Modified Early Warning Signs - Sepsis Recognition Score
MS: Mental status
NT-proBNP: N-terminal pro-brain natriuretic peptide
RAP: Rapid recovery
SCIRC: Sepsis and Critical Illness Research Center
SD: Standard deviation
SE: Standard error
SF-36: Short Form-36
SPPB: Short physical performance battery
